# Mr. Fuge, on Cow-Pox

**Published:** 1802-01

**Authors:** 


### POSTSCRIPT.

MR. FUGE, a fellow-labourer in the ſame field, and who fills the very important ſituation of Firſt Surgeon of the Royal Naval Hoſpital at Stonehouſe with diſtinguiſhed zeal and ability, has juſt now favoured me with the following highly intereſting communication; and I am much obliged to Mr. Fuge for the handſome manner of conveying it.

"My

"My dear Sir, *Royal Hospital, Dec.* 8, 1801.

"I have great pleafure in communicating to you a circumftance ftrongly illuftrative of *the beneficial effects* of the Vaccine Inoculation. It furnifhes the moft unequivocal evidence of its benign and falutary influences, and muft be highly gratifying to a man, feelingly alive to the calls of humanity, and whofe active and benevolent zeal has prompted him, with fo much ability, to ftep forward in fupport of one of the greateft gifts ever conferred on mankind.

Initiated among the earlieft in the practice, I have had great reafon to be fatisfied with the proofs that have fallen under my own obfervation, againft the fubfequent agency of Variolous Inoculation; every impartial obferver muft know that its fuccefs has been conftant and invariable. I have known no inftance of its failure, nor have heard of any to which the leaft degree of credit could attach. I have only to lament, in common with the moft refpectable practitioners in thefe towns, that its importance is not duly appreciated. Averfe to all innovations, the public mind is not yet prepared to welcome fo great a boon. Truth, however, muft prevail; thofe prejudices will gradually fubfide, and its reputation be fixed on the moft permanent bafis.

Mary Rogers, aged twelve, was admitted into a charity fchool, at Plymouth, in July laft, founded by Dame Hannah Rogers, for the education and fupport of a limited number of girls. In the abfence of my fon I was defired to attend. She had been naturally weak, but was now fo reduced in ftrength, as in walking to drag one foot after the other; the mufcles of the neck feemed unequal to their ordinary exertions, and her chin actually refted on her breaft; belly tumid, quick pulfe, appetite near gone, a fallow wan countenance; indeed, a general emaciation and mufcular debility was extreme, and the whole appearance indicating *not the smallest hope of recovery.* Believing it to be a cafe of tabes mefenterica, and fufpecting worms, repeated mild dofes of merc. dulc. and rhubarb were exhibited, interpofing chalybeats and other tonics, with a generous diet of animal food, wine, and porter, with fuch other auxiliary means as the cafe feemed to require. No worms were difcharged, nor the fmalleft advantage gained, in more than fix weeks; at which time, having learned that fhe never had the fmall-pox; knowing, alfo, that Vaccinia, when properly treated, leaves nothing injurious to the conftitution; and believing, on the contrary, that I had *more than once* feen health improved by paffing through the Vaccine Difeafe, I propofed *(as my dernier resort)* to vaccinate her. The operation took place about the middle of September; it had all the characteriftic istic marks of legitimate disease in its progress and termination, with scarcely a perceptible constitutional affection. Subsequent to the Vaccine, in the following month, she was subjected to the variolous infection, with no more than an accompanying local inflammation. By this time *(all medicines being discontinued* from the period of her first inoculation) her health was in a state of progressive improvement; and though a fever of the typhoid kind affected many others in the school, threatening to become general, she only felt a trifling indisposition for two days, and is now in the possession of *good health*.

"The master, who is an intelligent man, is become a zealous promoter of the new inoculation, and upon all occasions extremely eloquent in praise of it, will feel happy to add his testimony whenever called upon. My present avocation, and your desire to be put in immediate possession of this fact, will not allow me time to venture a conjecture on so remarkable a change superinduced in the system. The cause may for a while continue inexplicable; but insusceptibility of vaccinated patients to receive the variolous infection is a truth that must eventually triumph over every opposition. Believe me, most sincerely,

Your's, &c.
SAMUEL FUGE."

*To Mr. Dunning, Surgeon, Dock.*

The preceding valuable case, with some others which I can adduce, and shall I hope soon offer the public, tend to countenance a conjecture which with great deference I threw out in my Observations on the Cow-pox, That possibly this benign yet powerful agent might be employed, with some faint prospect of success, in scrofula and incipient cases of phthisis; and to encourage even a wish, that as an auxiliary, the *Aura Vaccina of Vaccination* might be admitted, with the *Aura Vaccina of the cow-house*, on the list of those new and strongly combined forces, which the indefatigable BEDDOES is continually bringing up against these insidious and fatal enemies of life. The Doctor has, with great independence of mind, levelled a blow at the shackles and unavailing slavery of routine practice, (in those diseases at least) which will shake its foundations; and having also, on all occasions, taught that the province of medicine must be a great and generous science, never a contending trade, seems already to have deserved that the epitaph, which he himself has so energetically drawn, should hereafter be engraven on his own tomb:

"Sed iterum, iterumque precor, quod redeat serus in coelis."

